# The phosphorylation of HIV-1 Gag by atypical protein kinase C facilitates viral infectivity by promoting Vpr incorporation into virions

**DOI:** 10.1186/1742-4690-11-9

**Published:** 2014-01-22

**Authors:** Ayumi Kudoh, Shoukichi Takahama, Tatsuya Sawasaki, Hirotaka Ode, Masaru Yokoyama, Akiko Okayama, Akiyo Ishikawa, Kei Miyakawa, Satoko Matsunaga, Hirokazu Kimura, Wataru Sugiura, Hironori Sato, Hisashi Hirano, Shigeo Ohno, Naoki Yamamoto, Akihide Ryo

**Affiliations:** 1 Department of Microbiology, Yokohama City University School of Medicine, Yokohama, Kanagawa, Japan; 2 Venture Business Laboratory, Ehime University, Matsuyama, Ehime, Japan; 3 Proteo-Science Center, Ehime University, Matsuyama, Ehime, Japan; 4 Clinical Research Center, National Hospital Organization Nagoya Medical Center, Nagoya, Aichi, Japan; 5 Pathogen Genomics Center, National Institute of Infectious Diseases, Musashi Murayama, Tokyo, Japan; 6 Supramolecular Biology, International Graduate School of Arts and Sciences, Yokohama City University, Yokohama, Kanagawa, Japan; 7 Infectious Disease Surveillance Center, National Institute of Infectious Diseases, Musashi Murayama, Tokyo, Japan; 8 Department of Molecular Biology, Yokohama City University School of Medicine, Yokohama, Kanagawa, Japan; 9 Department of Microbiology, National University of Singapore, Singapore, Singapore

**Keywords:** HIV-1 infection, Phosphorylation, Vpr, aPKC

## Abstract

**Background:**

Human immunodeficiency virus type 1 (HIV-1) Gag is the main structural protein that mediates the assembly and release of virus-like particles (VLPs) from an infected cell membrane. The Gag C-terminal p6 domain contains short sequence motifs that facilitate virus release from the plasma membrane and mediate incorporation of the viral Vpr protein. Gag p6 has also been found to be phosphorylated during HIV-1 infection and this event may affect virus replication. However, the kinase that directs the phosphorylation of Gag p6 toward virus replication remains to be identified. In our present study, we identified this kinase using a proteomic approach and further delineate its role in HIV-1 replication.

**Results:**

A proteomic approach was designed to systematically identify human protein kinases that potently interact with HIV-1 Gag and successfully identified 22 candidates. Among this panel, atypical protein kinase C (aPKC) was found to phosphorylate HIV-1 Gag p6. Subsequent LC-MS/MS and immunoblotting analysis with a phospho-specific antibody confirmed both in vitro and in vivo that aPKC phosphorylates HIV-1 Gag at Ser487. Computer-assisted structural modeling and a subsequent cell-based assay revealed that this phosphorylation event is necessary for the interaction between Gag and Vpr and results in the incorporation of Vpr into virions. Moreover, the inhibition of aPKC activity reduced the Vpr levels in virions and impaired HIV-1 infectivity of human primary macrophages.

**Conclusion:**

Our current results indicate for the first time that HIV-1 Gag phosphorylation on Ser487 is mediated by aPKC and that this kinase may regulate the incorporation of Vpr into HIV-1 virions and thereby supports virus infectivity. Furthermore, aPKC inhibition efficiently suppresses HIV-1 infectivity in macrophages. aPKC may therefore be an intriguing therapeutic target for HIV-1 infection.

## Background

Human immunodeficiency virus type 1 (HIV-1), a causative agent of AIDS, is an intracellular parasite that has evolved to invade complex human systems and utilize its host machinery for its proliferation. A dynamic interplay between HIV-1 and its human host systems plays a crucial role in promoting virus replication. The identification of the host factors required for viral infection can provide further insights into the nature of HIV-1 replication pathways and assist with identifying new targets for anti-viral therapies. Recent studies have revealed that host factors are involved in the post-translational modification of viral proteins, such as phosphorylation and ubiquitination, thereby regulating HIV-1 replication and pathogenicity [1-3].

The *gag* gene of HIV-1 encodes both structural and functional proteins essential for the assembly and release of enveloped virus-like particles [4]. In the infected cell, Gag is synthesized as a 55-kDa polyprotein and assembled into spherical immature particles at plasma membrane. Concomitant with, or after these viral particles pinch off and are released from the host cell via budding, the virus-encoded protease becomes activated and cleaves Gag into its functional subdomains, matrix (MA, p17), capsid (CA, p24), and nucleocapsid (NC, p7), as well as several shorter segments: SP1 (spacer peptide 1), SP2, and p6. This proteolytic maturation in tandem with the incorporation of viral enzymes and accessory proteins into virions results in the acquisition of HIV-1 infectivity [5-8].

Retroviral assembly can be subdivided into distinct stages of Gag membrane targeting, virus bud formation and induction of membrane curvature, and release of the newly assembled virus bud through a membrane fission event. HIV-1 budding from the cell surface depends on viral late domains within Gag p6 [9]. Two late domains have been identified within p6, the PTAP and LYPX_n_L motifs. The PTAP motif binds the cellular protein Tsg101 [10,11], whereas the LYPX_n_L motif is the docking site for Alix/AIP-1 [12,13]. Tsg101 functions in HIV-1 budding as a member of the Endosomal Sorting Complex Required for Transport-1 (ESCRT-I), which initiates the sorting of surface proteins into late endosomal compartments known as multivesicular bodies (MVB) [14,15]. Alix, ALG-2 interacting protein, functions in endosomal metabolism, promotes viral budding by interconnecting HIV-1 Gag with the ESCRT-III CHMP4 proteins [16,17].

Another important domain within Gag p6 is the C-terminal LXXLF domain. Interestingly, both the Leu486 and Leu491 residues in this motif are highly conserved and together with the downstream Phe492, comprise the LXXLF binding domain for the HIV-1 accessory viral protein R (Vpr) [18,19]. The substitution of residues in this domain causes a decrease in the Vpr incorporation levels compared with full-length HIV-1 Gag protein, indicating that this conserved region is essential for this process.

HIV-1 Vpr is a non-structural protein that is incorporated into the viral particles and possesses several characteristic features that are known to play important roles in HIV-1 replication and disease progression. Vpr mediates multiple functions, including the nuclear import of the HIV-1 pre-integration complex, G2 cell cycle arrest, the transactivation of both viral replication and host genes, and the induction of apoptosis [20]. Vpr interacts with the LXXLF binding domain of Gag p6 and is thereby packaged into the virus particles. Virion-incorporated Vpr is known to positively regulate the infection of non-dividing cells and enhance virus production in macrophages and in resting T cells. However, it remains elusive whether and how Vpr incorporation is indeed regulated. Furthermore, although p6 has been shown to be post-translationally modified by phosphorylation [2,21,22], it is unknown whether this phosphorylation event has any functional relevance to Vpr incorporation and HIV-1 infectivity.

In our current study, we utilized an in vitro high-throughput protein-protein interaction assay using full-length HIV-1 Gag and host protein kinases synthesized by the wheat germ cell-free protein production system in an attempt to identify the kinase (s) that directs the phosphorylation of Gag p6 to promote virus replication. We here report that atypical protein kinase C (aPKC) is a functional interactor of HIV-1 Gag and facilitates viral infectivity by promoting the incorporation of Vpr into virions. We provide evidence that Gag Ser487 (p6 Ser40) is phosphorylated by aPKC, and that this phosphorylation is essential for p6-Vpr interactions and the resultant Vpr incorporation within viral particles. Using computer-assisted structural modeling, we further explore the biological significance of the phosphorylation of Gag-p6 Ser487 by aPKC for the physiological interaction between Gag and Vpr. Our current study sheds new light on the molecular link between Gag phosphorylation and viral infectivity through the incorporation of Vpr into virions.

## Results

### aPKC binds and phosphorylates HIV-1 Gag

**Figure 1:**
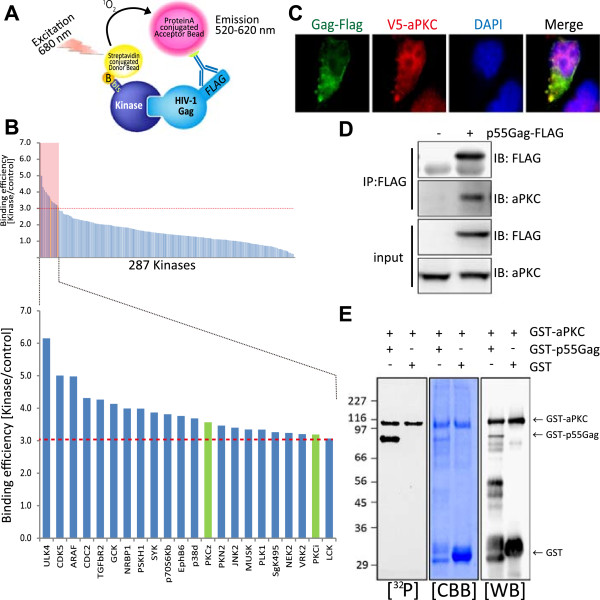
**aPKC phosphorylates HIV**-**1 Gag. (A)** Schematic representation of the AlphaScreen based luminescent system used to screen for human kinases that interact with HIV-1 Gag. Briefly, HIV-1 Gag was incubated with each human protein kinase and protein A-conjugated acceptor beads with anti-FLAG antibody and streptavidin coated donor beads were added and bound to the tagged substrate. Upon laser excitation, the donor beads convert ambient oxygen to singlet oxygen. When a molecular interaction occurs between HIV-1 Gag and a particular kinase, singlet oxygen transfers across to activate the acceptor beads and subsequently emit light at 520–620 nm. **(B)** HIV-1 Gag interacting kinases identified from a human kinase protein library. **(C)** Confocal microscopy analysis of V5-aPKC (Alexa 568) and Gag-Flag (Alexa 488) in 293T cells. **(D)** Immunoprecipitation of Gag with aPKC. 293T cells were transfected with Flag tagged Gag and cell lysates were immunoprecipitated with anti-Flag antibody 48 hours later. Samples were then probed with anti-aPKC and anti-Flag antibodies. Bottom panel shows input for pull-down assay. **(E)***In vitro* phosphorylation of HIV-1 Gag by aPKC. GST, GST tagged aPKC and Gag proteins were expressed and affinity-purified from wheat germ cell-free extract. aPKC-mediated phosphorylation was assessed by incubating recombinant GST or GST-Gag with recombinant active GST-aPKC in the presence of [γ-^32^P] ATP. The reaction products were analyzed by autoradiography, Coomassie Brilliant Blue (CBB) stain and blotted with GST antibody.

Our initial goal was to identify host kinases that phosphorylate the HIV-1 Gag protein. Because Gag phosphorylation is important for its functional role, we focused on human protein kinases as potential Gag regulators. We synthesized more than 287 full-length protein kinases using a wheat germ cell-free protein production system, and screened them for their association with Gag with the amplified luminescent proximity homogenous assay (AlphaScreen) [23]. In this method, the extent of the protein-protein interaction was measured by assaying the luminescence intensity (Figure 1A). Full-length Gag and human protein kinases were synthesized using a wheat germ cell-free system and subjected to an AlphaScreen assessment. The binding efficiency of HIV-1 Gag with each kinase was normalized relative to the luminescent activity of a control DHFR protein (Figure 1B). When a relative light unit per cutoff (RLU/Co) ratio of ≥ 3.0 was used as the threshold, we found that 22 host kinases could selectively interact with HIV-1 Gag and thus were identified as primary kinase candidates for the phosphorylation of HIV-1 Gag (Figure 1B).

Our assay detected Erk2 and PKCβ as Gag interactors (S/N = 1.76 and 1.17, respectively), both of which have been already reported to phosphorylate Gag during HIV-1 infection [2,22,24,25]. This validated our screening approach. Interestingly, we further found that the aPKC family kinases, PKCζ and PKCι, could interact with HIV-1 Gag at a relatively high score (S/N = 3.57 and 3.19, respectively). PKCζ and PKCι share a more than 70% amino acid identity in entire protein sequence and 84% in the catalytic domain, and an almost identical substrate specificity [26]. We thus focused on aPKC as a previously uncharacterized Gag-interacting factor for further in depth functional analysis.

To better understand the functional relevance of aPKC in HIV-1 infection, we first examined the subcellular localization of both HIV-1 Gag protein and aPKC protein in 293T cells by immunofluorescent analysis. 293T cells were transfected with Flag tagged HIV-1 Gag and V5-aPKC expression vector. Gag-Flag displayed a punctate expression pattern in the cytoplasm and a partial co-localization with aPKC in cytoplasm and plasma membrane (Figure 1C).

We performed immunoprecipitation analysis and found that aPKC could bind Gag in cells (Figure 1D). We next examined whether aPKC can directly phosphorylate HIV-1 Gag protein in vitro. Recombinant GST-Gag or GST proteins were expressed and purified from wheat germ cell-free extract by glutathione sepharose beads and used as substrates for in vitro kinase assays. aPKC was found to phosphorylate GST-Gag but not GST, with a prominent auto-phosphorylation of aPKC also observed (Figure 1E). These data together indicate that aPKC binds and phosphorylates HIV-1 Gag.

### aPKC phosphorylates the Ser487 residue of HIV-1 Gag

**Figure 2:**
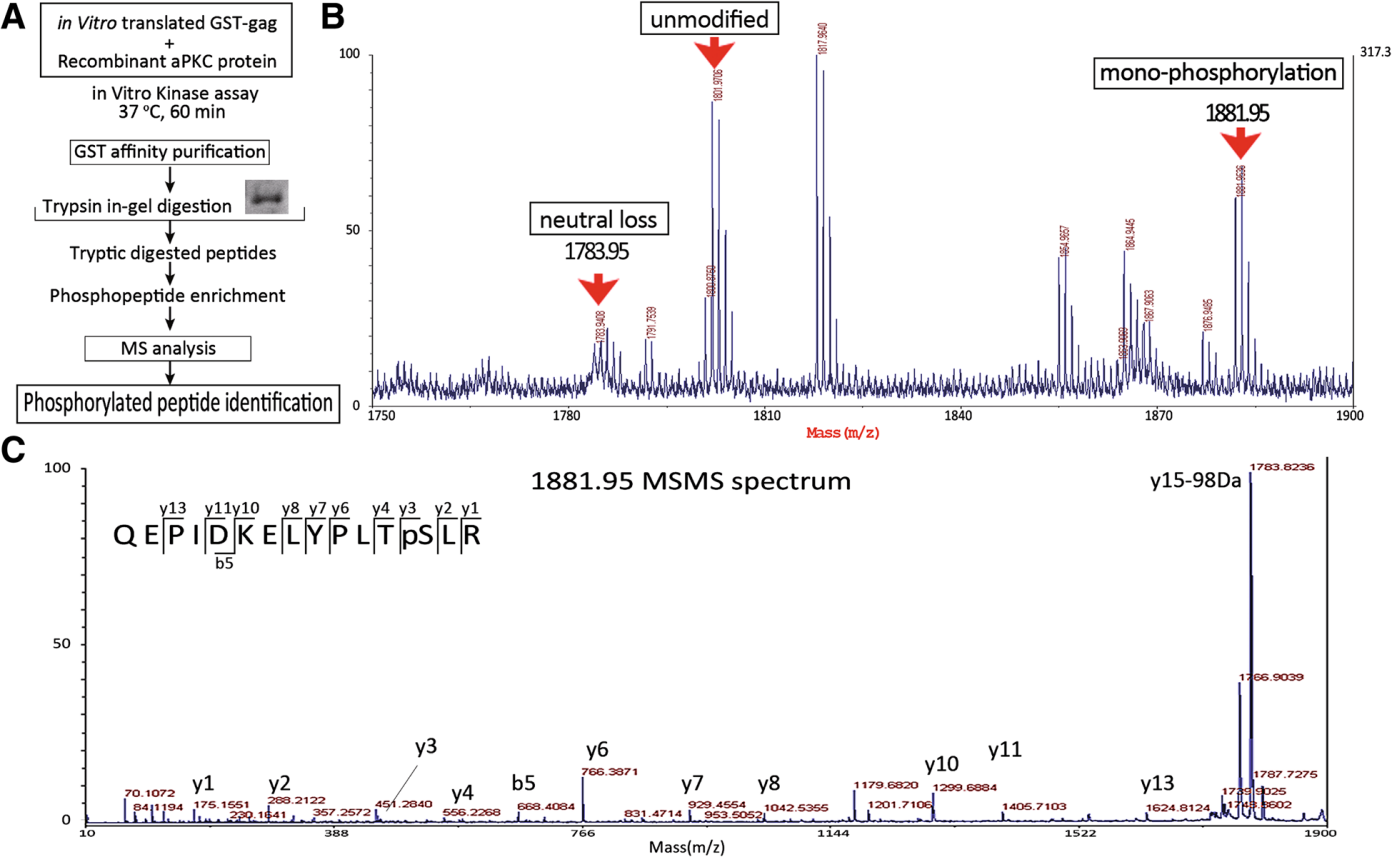
**Identification of the phosphorylated Gag**-**p6 site. (A)** Schematic overview of the experimental workflow. **(B)** MS peptide map of phosphorylated GST-Gag after trypsin digestion. The red arrow indicates the 1881.95 Da peak which is unique to the sample treated with active aPKC. This is consistent with the mass of the theoretical phosphorylated peptides and the map shows a neutral loss peak at 1783.95. **(C)** The resulting phosphopeptide was analyzed using MALDI-TOF/TOF MS. Fragment ions of the b and y-series identified in the tandem mass spectra from the peptide are shown. Tandem mass spectra of m/z 1881.95 signals revealed that each sequence corresponded to the phosphopeptide (QEPIDKELYPLTpSLR) of Gag-P6.

We next sought to determine the sites of aPKC phosphorylation in HIV-1 Gag. GST-Gag was incubated with recombinant aPKC for their phosphorylation and this mixture was then processed for proteomic analysis. Initial phosphorylation site analysis was performed using the data dependent of tandem matrix-assisted laser desorption Ionization-time of flight mass spectrometry (MALDI-TOF/TOF-MS), followed by in depth analysis with selected peptides through data collection. Fragmentation of this peptide by MS/MS produced a spectrum through which we identified one of the b-ions and 10 of the y-ions matching the sequence QEPIDKELYPLTpSLR. Tandem mass spectra of the signals at m/z 1881.95, m/z 1783.95(neutral loss) and m/z 1801.97 revealed sequences corresponding to the unmodified, mono- phospho peptide of Gag-p6 (QEPIDKELYPLTpSLR; Figure 2). Furthermore, a Mascot search result identified the sequence QEPIDKELYPLTpSLR (score 73). The Ser487 site was found to be located at Ser40 of Gag-p6 domain in close proximity to both LYPX_n_L and LXXLF motif.

**Figure 3:**
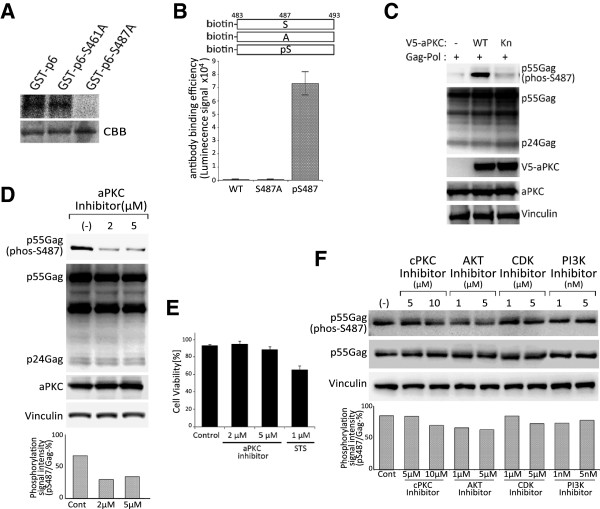
**aPKC phosphorylates HIV**-**1 Gag on Ser487. (A)** GST-p6, GST-p6S461A or GST-p6S487A proteins were expressed and affinity-purified from wheat germ cell-free extracts. aPKC-mediated phosphorylation was assessed by incubating recombinant GST-p6, GST-p6S461A or GST-p6S487A with recombinant active GST-aPKC in the presence of [γ-^32^P] ATP for 60 min at 37°C. The reaction products were analyzed by autoradiography and Coomassie Brilliant Blue (CBB) staining. **(B)** Assessment of phospho-Ser487 antibody specificity and binding efficiency using the AlphaScreen system. Biotin tagged peptides were incubated with phospho-Ser487 specific antibodies for. The graph shows the luminescence signal intensity which represents antibody binding efficiency. Each value is the mean of three independent experiments. **(C)** 293T cells were transfected with wild type Gag-pol and either V5 tagged wild type aPKC (WT) or kinase-negative aPKC (Kn). Whole-cell extracts were prepared, and equal amounts of proteins for each sample were separated by SDS-PAGE and subjected to western blot analysis using indicated antibodies. **(D)** Effects of aPKC inhibitor on the phosphorylation of Gag-Ser487. 293T cells were transfected with Gag-pol expression vector and cultured in the presence of the indicated concentrations of aPKC inhibitor. Clarified cell lysates were prepared and equal amounts of proteins were subjected to SDS-PAGE, followed by western blot analysis using indicated antibodies. Densitometry analysis of the p55Gag (pS487)/p55Gag bands in the western blot is shown in the bottom panel. **(E)** Effects of aPKC inhibitor on viability of 293T cells. 293T cells were treated with aPKC inhibitor (2 or 5 μM) or 1 μM Staurosporine (STS) and analysed for cell viability using trypan blue exclusion at 48 hours. **(F)** Effects of kinase specific inhibitors on the phosphorylation of Gag-Ser487. 293T cells were transfected with Gag-pol expression vector and treated with inhibitors against cPKC, AKT, CDK and PI3K. Cell lysates were prepared and analyzed as described in **(D)**.

Based on our MS analysis, we constructed a GST-tagged p6 and its site-directed mutant GST-p6-Ser487Ala (S487A) and GST-p6-Ser461Ala (S461A) as a negative control. Subsequent in vitro kinase assay results demonstrated that GST-p6 is phosphorylated by aPKC, but not GST-p6-S487A (Figure 3A). These results suggested that aPKC indeed phosphorylates the Ser487 residue of HIV-1 Gag *in vitro*.

To further assess the phosphorylation of Gag at Ser487, we generated a polyclonal antibody against phosphoryated-Ser487 (pS487). We initially confirmed the specificity and sensitivity of the antibody using the AlphaScreen system. We found that our antibody recognized only Ser487 phosphorylated peptides but neither a non-phosphorylated peptide nor a peptide harboring a Ser487 to Ala substitution (Figure 3B). We then used this antibody for in depth cell culture study. 293T cells were transfected with V5 tagged wild type aPKC or a kinase-negative mutant (aPKC-Kn), together with wild type Gag-Pol. A marked increase in the level of Gag phosphorylation at Ser487 was observed in cells expressing the wild type aPKC, whereas there was no obvious increase in the amounts of phosphorylation in either aPKC-Kn or mock transfected cells (Figure 3C). These observations clearly indicate that the expression of aPKC leads to the phosphorylation of HIV-1 Gag at Ser487 in cells, and that this phosphorylation is dependent of the kinase activity of aPKC.

To further investigate whether the phosphorylation of HIV-1 Gag at Ser487 is mediated by endogenous aPKC activity, we employed a myristoylated PKCζ pseudosubstrate peptide as an aPKC inhibitor. This PKCζ pseudosubstrate peptide mimics the substrate binding site in PKCζ (113–125) and PKCι (114–126), and suppresses the activity of endogenous PKCι and PKCζ. HIV-1 Gag-Pol expression plasmids were transfected into 293T cells with or without aPKC inhibitor treatment. Immunoblot analysis revealed that the aPKC inhibitor suppressed Gag phosphorylation at Ser487. Subsequent titration analysis demonstrated a dose-dependent inhibitory effect of the PKCζ pseudosubstrate peptide by showing an 74.9% and 70.4% decrease in Gag phosphorylation at 2 μM and 5 μM doses, respectively (Figure 3D). Note that at these concentrations the aPKC inhibitor did not affect the expression levels of endogenous aPKC as well as a house-keeping protein Vinculin (Figure 3D). Furthermore, cell viability was not prominently affected by aPKC inhibitor when cells were assessed by trypan blue exclusion (Figure 3E). Conventional PKC (PKCα, PKCβ), Akt, CDK and PI3 kinases have been reported previously to affect HIV-1 replication through their phosphorylation of HIV-1 or of host proteins [3,24,27-30]. We thus also investigated using specific inhibitors whether these kinases could mediate the phosphorylation of HIV-1 Gag at Ser487. Our results show that neither PKCα nor PKCβ specific pseudosubstrates affect Gag phosphorylation at Ser487 (Figure 3F). Similarly, neither Akt inhibitor, the CDK inhibitor roscovitine nor the PI3K inhibitor wortmannin blocked Gag phosphorylation at Ser487 (Figure 3F). Taken together, these observations indicate that aPKC specifically phosphorylates HIV-1 Gag at Ser487 both *in vitro* and *in vivo*.

### The phosphorylation of Gag Ser487 facilitates the interaction between Gag and Vpr

**Figure 4:**
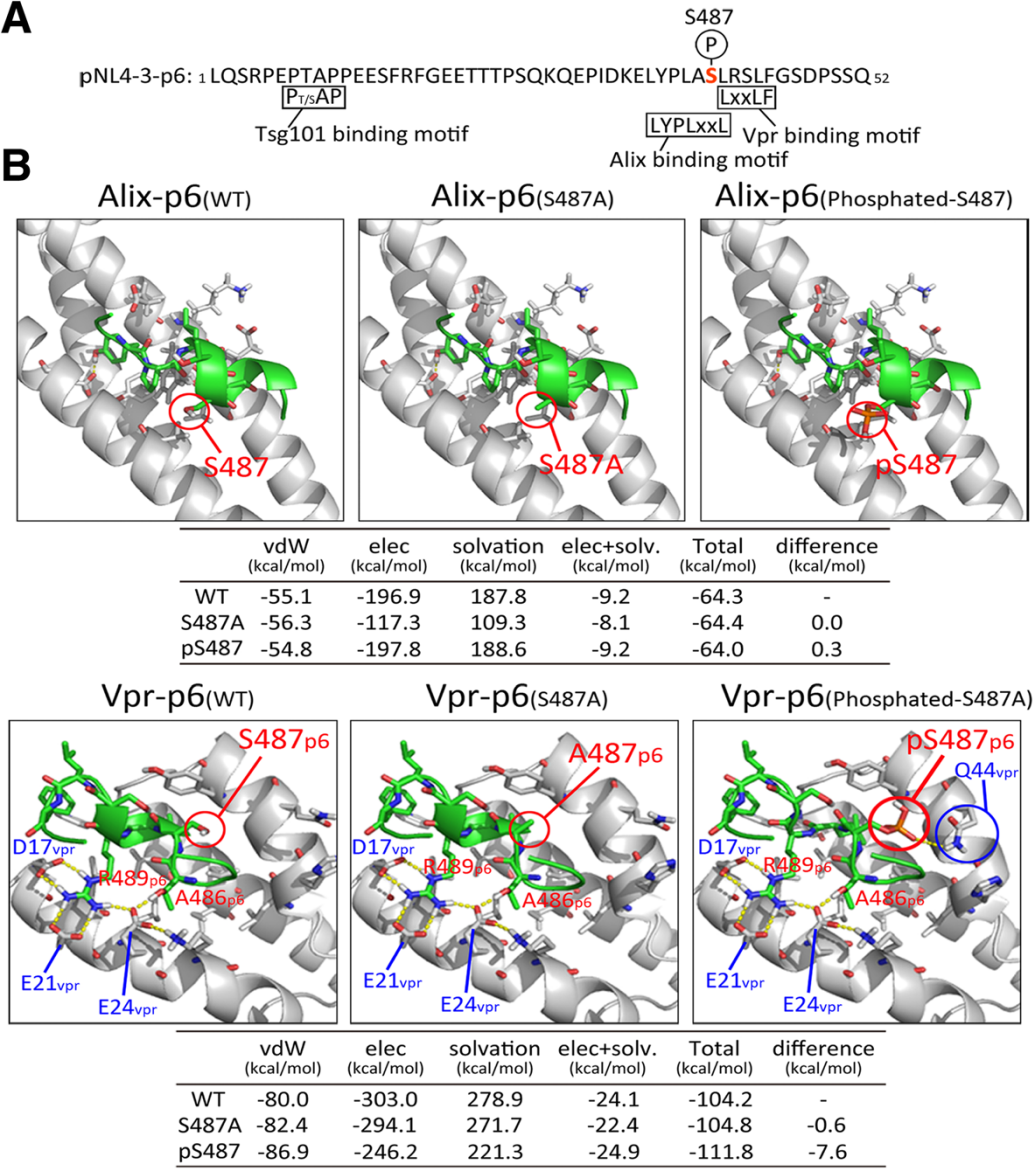
**The phosphorylation of Gag at Ser487 facilitates the interaction of Vpr and Gag**-**p6. (A)** Schematic representation of the HIV-1 Gag-p6 sequence indicating the binding motifs, Tsg101, Alix and Vpr, located around the Ser487 site. **(B)** Complex structure models of Gag-p6 with Alix or Vpr. The structural calculations were undertaken using MOE. Upper panel shows structural models of the interaction of Alix with wild-type Gag-p6, Gag-p6Ser487A or phosphorylated Gag-p6-Ser487. Bottom panel shows structural models of the interaction of Vpr with wild-type Gag-p6, Gag-p6Ser487A or phosphorylated Gag-p6-Ser487. Binding energies of p6 with Alix or Vpr calculated from the structural models were shown in the bottom tables, where the highly negative value indicates the stable binding.

HIV-1 Gag p6 contains a late domain consisting of three protein binding motifs, PTAP (Tsg101 binding), LYPXnL (Alix binding) and C-terminal Vpr. Ser487 is located in the Alix binding motif and is also adjacent to the Vpr binding motif spanning amino acids 488–492 (Figure 4A). To obtain structural-based information on Gag phosphorylation on Ser487 and how it affects the interaction of Gag with Alix or Vpr, we conducted computer-assisted molecular modeling of the Gag p6 domain coupled with peptides derived from either Alix or Vpr. The models constructed in this study included unphosphorylated and phosphorylated Gag-p6 (amino acids Tyr483-Ser494), and its Ser/Ala substituted mutant on Ser487 (S487A). Molecular modeling calculations with thermodynamically optimized three dimensional structures showed less than 1 Å of positional shifts of Cα atoms of Gag-p6 by phosphorylation, suggesting no obvious difference in the basic structure of Gag-p6 irrespective of the phosphorylation status. Furthermore, binding interface between Gag-p6 and Alix was not affected by the phosphorylation (Figure 4B, Upper panels) or Ser/Ala substitution of Gag Ser487 [31-33]. On the other hands, the binding of Gag-p6 with Vpr was facilitated since the phosphorylation of Ser487 can create another hydrogen bond between Gag-p6 and Vpr (Figure 4B, Lower panels). The Ser487 was predicted to form no hydrogen bonds with Vpr in non-phosphorylated state, whereas the phosphorylated Ser487 could form the hydrogen bond with Gln44 of Vpr. Consequently, binding energy calculated with Molecular Operating Environment (MOE) was significantly increased by phosphorylation of Ser487 only for the Gag-p6-Vpr complex (Figure 4B, tables). These data suggest that the phosphorylation of Gag-p6 on Ser487 could indeed affect the binding affinity of Gag-p6 with Vpr but not Alix.

**Figure 5:**
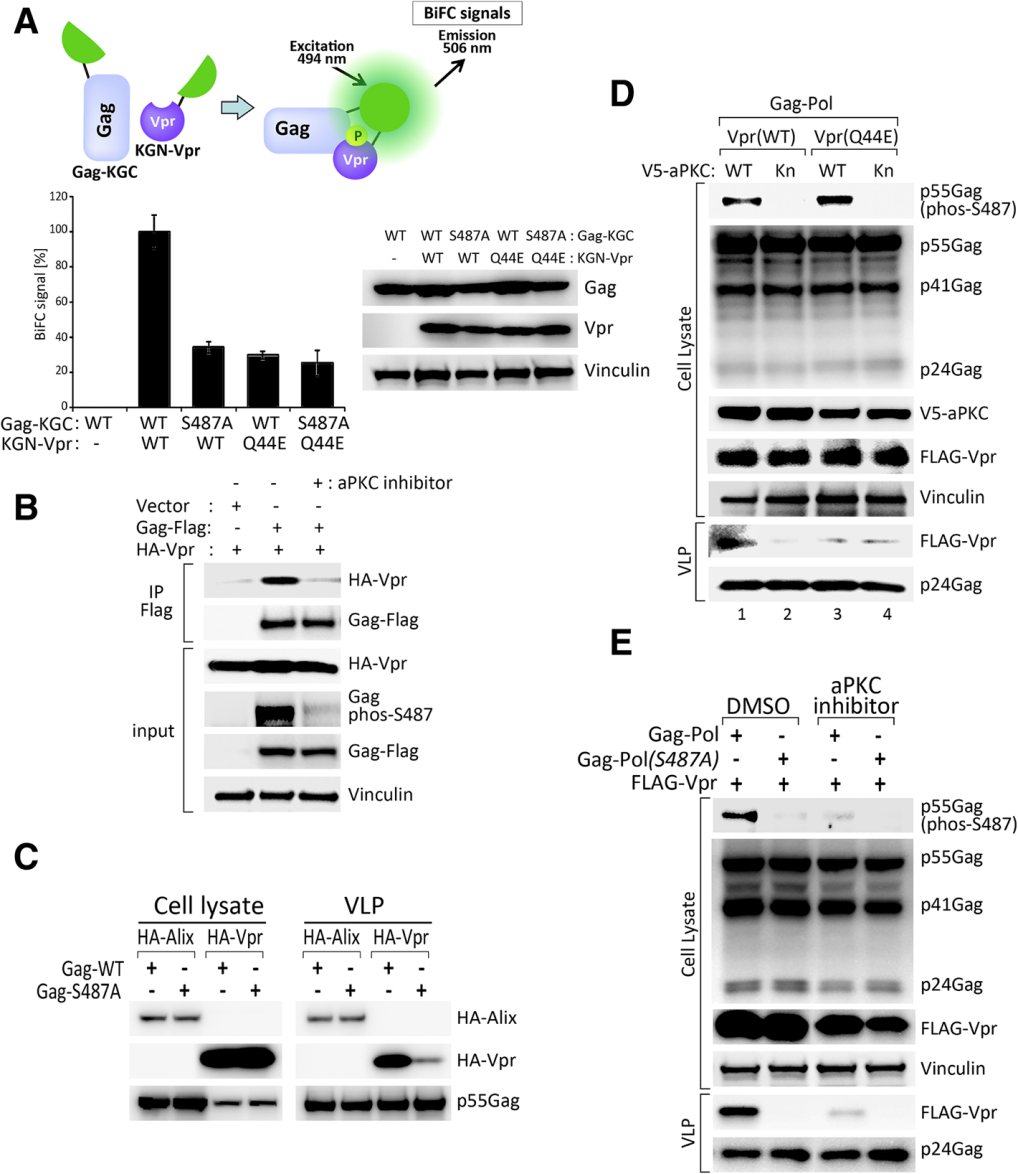
**The phosphorylation of Gag at Ser487 promotes the incorporation of Vpr into HIV**-**1 virions. (A)** Bimolecular fluorescence complementation (BiFC) of 293T cells expressing Gag-KGC and KGN-Vpr (upper panel). 293T cells were transfected with KGN-Vpr (WT or Q44E) and Gag-KGC or Gag (S487A)-KGC, were harvested and analyzed by flow cytometry to determine the BiFC signal at 48 hours post-transfection. Results represent the means of three independent experiments. The cells were harvested and subjected to western blot analysis. **(B)** The phosphorylation of Gag at Ser487 promotes interaction of Vpr with Gag. HA-tagged Vpr was co-transfected with Flag tagged Gag into 293T cells in presence of 5 μM aPKC inhibitor or DMSO, followed by pulldown with Flag-beads and immunoblotting with indicated antibodies. Bottom panel shows input for pulldown assay. **(C)** Differential effect of Gag Ser487 phosphorylation on Alix and Vpr incorporation into VLPs. 293T cells were transfected with GFP-tagged Gag (Gag-WT) or with the Ser487 point mutated Gag (Gag-S487A), with either HA-tagged Alix, or HA-tagged Vpr expression vectors. Cells and VLPs were collected at 24 hours post-transfection, and their protein extracts were resolved using SDS-PAGE and subjected to western blot analysis. **(D)** aPKC-mediated Gag phosphorylation at Ser487 facilitates Vpr incorporation into virions. 293T cells were transiently transfected with Gag-Pol, either Flag-tagged Vpr (WT) or Gln44 point mutated Vpr (Q44E), and with either V5-tagged aPKC (WT) or kinase-negative aPKC (Kn). Cell lysates and VLPs were collected 24 hours post-transfection and subjected to western blot analysis. **(E)** The phosphorylation of Gag-pol-Ser487 is required for Vpr incorporation into HIV-1 virions. 293T cells were transfected with Flag-tagged Vpr and either with Gag-Pol (WT) or with the Ser487 point mutated Gag-Pol (S487A). The transfected 293T cells were treated with DMSO or aPKC inhibitor for 24 hours. Cell lysates and VLPs were collected and subjected to western blot analysis.

Based on our structural modeling results, we next asked whether the phosphorylation of Gag at Ser487 has any effect on the interaction between Vpr and Gag. We have selected Bimolecular Fluorescence Complementation (BiFC) system to quantify the Vpr-Gag interaction in live cells as previously reported [34]. Plasmids encoding C-terminally KGC-tagged Gag (Gag-KGC) and N-terminally KGN-tagged Vpr (KGN-Vpr) were transfected and evaluated for BiFC signal by flow cytometry (Figure 5A). Flow cytometry analysis revealed that the interaction of Vpr with Gag-Ser487Ala mutant was reduced as compared with wild-type Gag (Figure 5A). To further assess whether the phosphorylation of Gag at Ser487 provides another hydrogen bond with Vpr Gln44 to facilitate Gag-Vpr interaction, we constructed Vpr Q44E mutant for BiFC analysis. Results demonstrated that Vpr Q44E mutant exhibited weker interactions to Gag and Gag S487A as compared with wild type Vpr (Figure 5A). We further found that aPKC inhibitor suppressed the interaction between Gag-Flag and HA-Vpr in imunoprecipitation analysis (Figure 5B).

### The phosphorylation of Gag at Ser487 affects Vpr incorporation into virions and viral infectivity

We next examined whether the phosphorylation of Gag at Ser487 has any effects on the incorporation of Vpr into HIV-1 virus like particles (VLP). As shown in Figure 4B, we found no distinct changes in the incorporation of Alix into VLPs regardless of a Ser/Ala substitution at Gag Ser487 in 293T cells. However, Vpr incorporation into VLP was significantly decreased in cells transfected with the Gag-Ser487Ala mutant as compared with cells transfected with wild-type Gag (Figure 5C). Hence, it is plausible that the phosphorylation of Gag at Ser487 may have an important role in its interaction with Vpr thereby affecting the Vpr incorporation into VLPs.

To further explore the relevance of Gag phosphorylation to HIV-1 replication, we examined whether aPKC kinase activity is necessary to regulate Vpr incorporation into HIV-1 virions. Gag phosphorylation at Ser487 was prominently enhanced by wild type aPKC but not kinase negative mutant aPKC (Kn) (Figure 5D). Concomitantly, the level of Vpr incorporation into virions was shown to be paralleled with the Gag phosphorylation status (lane 1 and 2 in Figure 5D). More importantly, virion incorporation of Vpr Q44E mutant was much lesser than wild-type Vpr irrespective of Gag phosphorylation at Ser487 (lane3 and 4 in Figure 5D). These results suggest that Gag phosphorylation at Ser487 is indeed affect Vpr incorporation and this process could be mediated by the Gln44 residue of Vpr. Although no significant effect of the Gag-pol S487A mutant on the Vpr expression levels in cells was evident, the Vpr incorporation level into VLPs was significantly reduced upon Gag-pol-S487Ala transfection (Figure 5E). Consistent with this result, the incorporation of Vpr into VLPs was significantly reduced in cells treated with the aPKC inhibitor peptide; the Vpr incorporation efficiency was reduced in aPKC inhibitor treated cells (Figure 5E). These data indicate that aPKC can enhance the incorporation of Vpr into HIV-1 virions.

**Figure 6:**
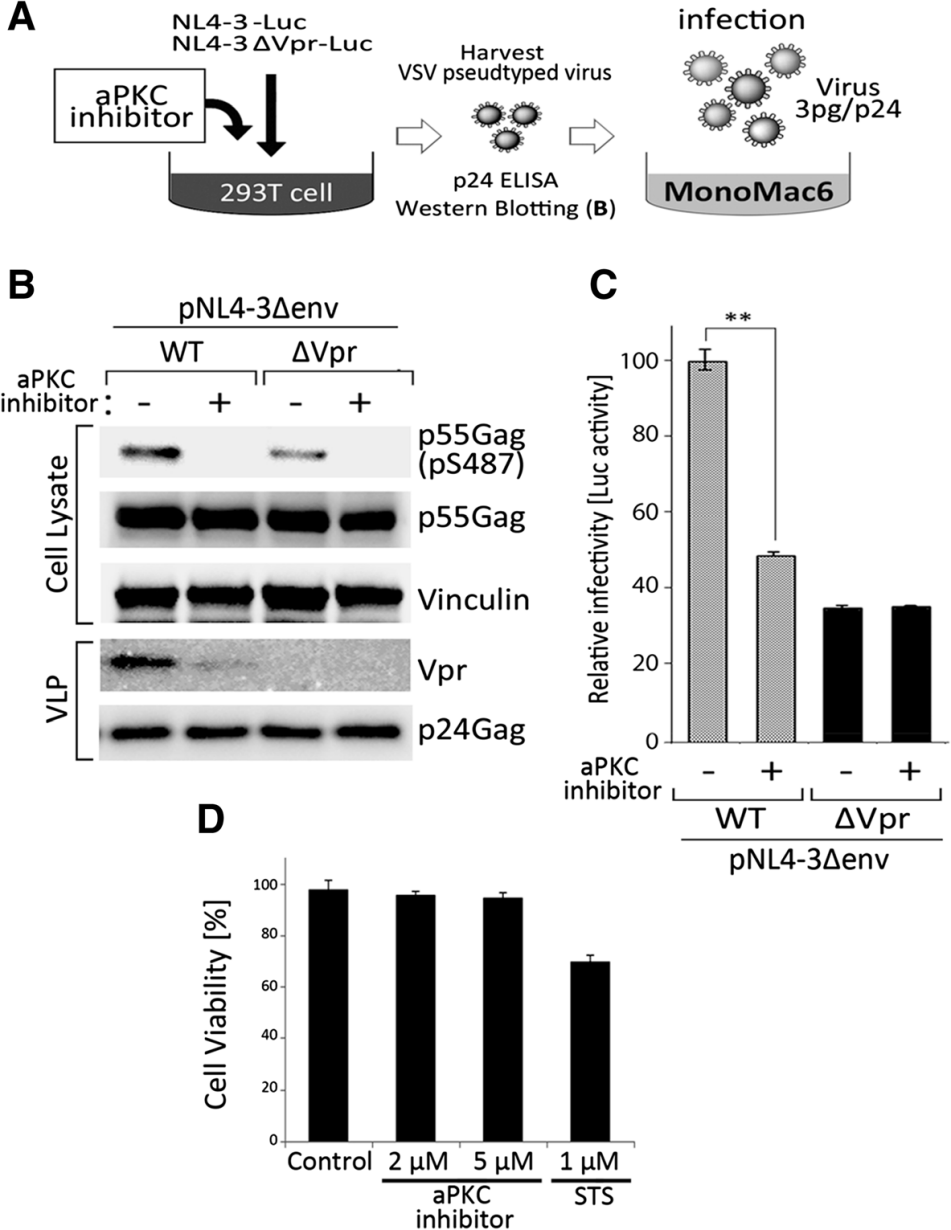
**The inhibition of aPKC significantly decreases single**-**round HIV**-**1 infection. (A)** Schematic representation of the experimental system. Briefly, 293T cells were either mock treated, or treated with aPKC inhibitor. After 4 hours, these cells were co-transfected with pNL4-3Δenv-luc or pNL4-3ΔenvΔVpr-luc and with pVSV-G. Viral release was measured through the quantification of p24CA antigen concentration in the culture supernatants at 48 hours post-transfection. MonoMac6 cells were infected with VSV-G pseudotyped WT and ΔVpr viruses for 48 hours. **(B)** The VSV-G pseudotyped WT and ΔVpr stocks generated from pNL4-3Δenv-luc and pNL4-3ΔenvΔVpr-luc with/without aPKC inhibitor treatment were analyzed by immunoblotting for the incorporation of Vpr into virion particles. **(C)** Viral infectivity was detected by measuring the luciferase activity in the cell lysates. Data are mean ± s.e.m. of three independent experiments. **(D)** Effects of aPKC inhibitors on viability of MonoMac6 cells. MonoMac6 cells were treated with aPKC inhibitor (2 or 5 μM) or 1 μM Staurosporine (STS) and analyzed for cell viability using trypan blue exclusion at 72 hours. Data are mean ± s.e.m. of three independent experiments: **p < 0.01, Student’s t-test.

It has been well established that Vpr incorporation into HIV-1 virions augments viral infectivity in macrophages [5,6,35-37]. We thus assessed whether aPKC affects HIV-1 infectivity by increasing Vpr incorporation into virions. We hypothesized that if the Gag phosphorylation at Ser487 by aPKC was beneficial for HIV-1 infection in this way, aPKC activity would affect wild type HIV-1 but not a Vpr-null virus. To test this, we employed pNL4-3ΔEnv-luc (WT) or pNL4-3ΔEnvΔVpr-luc (Vpr-null) strains. We then produced the corresponding viruses with a fusiogenic envelope G glycoprotein of the vesicular stomatitis virus (VSV-G) in the presence or absence of aPKC inhibitor in 293T cells (Figure 6A). Immunoblotting analysis of VLP demonstrated that the level of Vpr incorporation was prominently reduced by treatment with the aPKC peptide inhibitor (Figure 6B). The infectivity of the generated viruses was tested using the human monocyte/macrophage cell line MonoMac6. The aPKC inhibitor-treated WT virus exhibited approximately 50% less infectivity than the control WT virus (Figure 6C). The Vpr-null virus showed a 35% reduction in infectivity compared with the WT virus in the MonoMac6 cells (Figure 6C). However, the primarily low infectivity of the Vpr-null virus was not significantly affected by the aPKC inhibitor (Figure 6C). aPKC inhibitor did not exhibit obvious cytotoxic effect to MonoMac 6 cells (Figure 6D).

**Figure 7:**
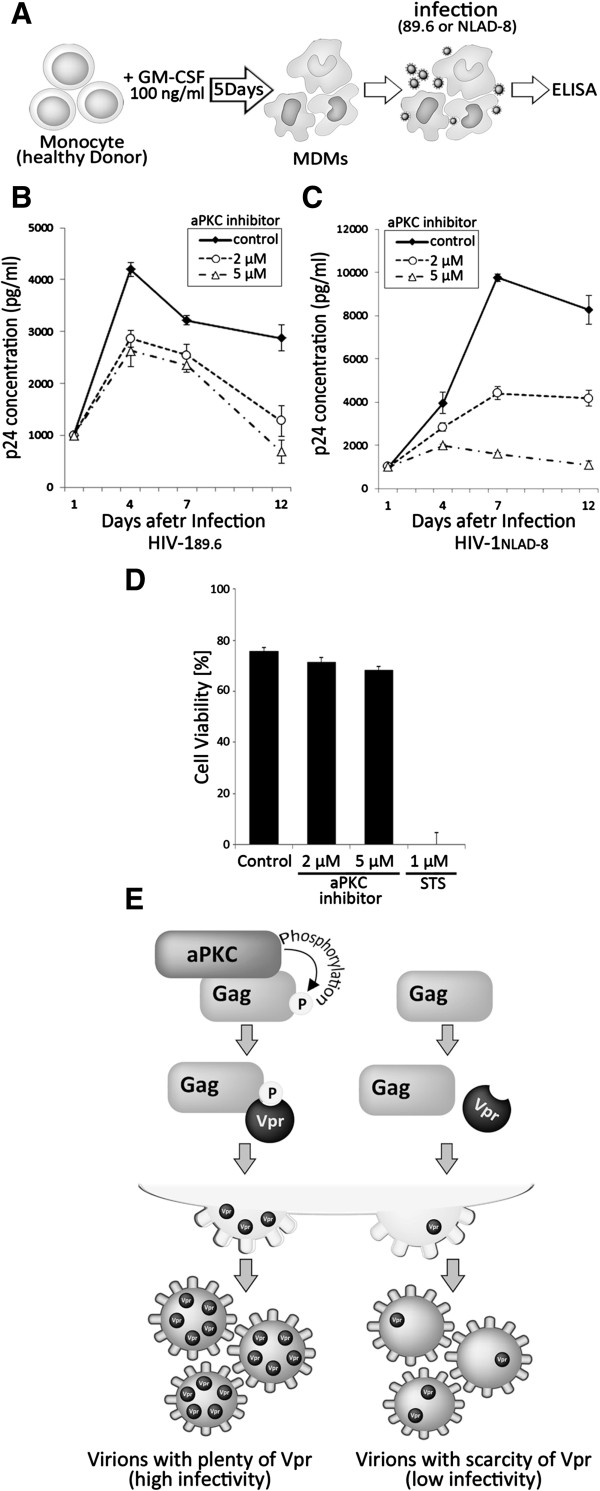
**The inhibition of aPKC significantly reduces replication competent HIV**-**1 infection. (A)** Schema of the experimental system using HIV-1 replication competent HIV-1_89.6_ and HIV-1_NLAD-8_. Monocytes were isolated from buffy coat from healthy blood donors by positive selection on Monocyte Enrichment Cocktail and density gradient centrifugation as described in Methods. MDMs were generated by culturing monocytes with 100 ng/ml granulocyte-macrophage colony-stimulation factor for 5 days before HIV-1 infection. MDMs were infected with 5 ng of p24 from **(B)** HIV-1_89.6_ or **(C)** HIV-1_NLAD-8_ virus and the levels of p24 capsid released into media during viral replication were assayed over a period of 12 days post infection. Data are the mean ± s.e.m. of three independent experiments: **P < 0.01, Student’s t-test. **(D)** Effects of aPKC inhibitor on viability of MDMs. MDMs cells were treated with aPKC inhibitor (2 or 5 μM) or 1 μM Staurosporine (STS) and analysed for cell viability using trypan blue exclusion at 96 hours. **(E)** Schematic model of our current study. aPKC phosphorylate HIV-1 Gag at Ser487. This phosphorylation promotes the interaction between Gag and Vpr, thereby facilitating viral infectivity in macrophages.

To assess the role of aPKC in multi-round HIV-1 replication in primary monocyte-derived macrophages (MDMs), we infected these cells with HIV-1_89.6_, a dual tropic virus, or HIV-1_NLAD8_, an R5 tropic virus, in conjunction with treatments of various concentrations of the aPKC inhibitor (Figure 7A). The results revealed that the aPKC inhibitor strongly suppressed the replication of both viruses in a dose-dependent manner (Figure 7B, C), although there was no obvious toxicity or growth inhibition in these cells (Figure 7D). Taken together, these results indicate that the phosphorylation of Gag by aPKC regulates Vpr incorporation and HIV-1 replication in macrophages.

## Discussion

We here demonstrate that aPKC is a crucial regulator of HIV-1 infection via the phosphorylation of Gag-p6 which enhances the incorporation of Vpr into virions. Our current data strongly suggest that Ser487 is the specific phosphorylation site on HIV-1 Gag for aPKC and is crucial for the Gag p6-Vpr interaction that leads to Vpr incorporation into viral particles. Furthermore, our current data demonstrate that an aPKC inhibitor prominently inhibits HIV-1 replication in primary human macrophages. Hence, the phosphorylation of Gag by aPKC may well be an important mechanism through which HIV-1 efficiently infects macrophages and by which an excessive accumulation of the cytotoxic Vpr protein in the host infected cells is prevented.

The Gag-p6 domain has been identified as the predominant site of phosphorylation in HIV-1 particles [22]. Ser487 is a highly conserved residue in this p6 domain among various HIV-1 strains, suggesting that the phosphorylation of this residue is of fundamental functional importance. Votteler et al. have demonstrated that a HIV-1 Gag mutant with a deleted PTAP region and a phenylalanine substitution at Ser487 (ΔPTAP/S487F) shows aberrant core formation and reduced viral infectivity in TZM-b1 cells [33]. More recently, steady state affinity analysis using a surface plasmon resonance sensorgram has revealed that the phosphorylated form of p6 at Ser487 has a stable binding affinity for cytoplasmic membranes [38]. These reports have therefore revealed that Gag Ser487 is a highly conserved phosphorylation site of likely crucial importance for HIV-1 infection. On the other hand, Radestock et al. recently reported in tissue culture experiments that the phosphorylation of Gag-p6 including Ser487 is dispensable for HIV-1 infectivity. These authors showed that asparagine substitutions at five serine residues (Ser487, Ser490, Ser494, Ser497 and Ser498) within the C-terminus of Gag p6 produced no impairment of Gag assembly or virus release and caused only very subtle deficiencies in viral infectivity in T-cell lines and in primary lymphocytes [39]. These discrepancies may be due to different experimental approaches using different Gag substitution mutants as well as different cell types. In contrast, our present approach is distinct from these earlier studies as we initially attempted to identify the kinases responsible for Gag-p6 phosphorylation and then explore their role in HIV-1 replication. Our current results clearly demonstrate that aPKC phosphorylates Gag-p6 and regulates the interaction of Gag with Vpr for the incorporation of Vpr into virus particles. These specific effects of aPKC-mediated Gag-p6 phosphorylation are consistent with the evidence that the substitution of Gag Ser487 for Ala significantly decreases Vpr incorporation and viral infectivity. On the other hand, inhibition of aPKC in cells may have other additional effects on HIV-1 replication cycle rather than Gag phosphorylation for the Vpr incorporation. To observe the specific effect of aPKC on Gag phosphorylation, we created Gag and Vpr mutants devoid of the effect of aPKC and these mutants were less competent in virus replication. However, aPKC may regulate other cellular function directing HIV replication. Although our current data clearly demonstrate a crucial role of aPKC in Gag Ser487 phosphorylation and interaction Gag-Vpr to Vpr incorporation, further detailed analyses may be necessary to clarify the molecular signature of Gag p6 phosphorylation on multiple stages of the HIV-1 replication cycle.

We show from our current experiments that Gag Ser487 phosphorylation has a significant impact on p6-Vpr binding. Vpr is a non-structural viral protein that is incorporated into virions and possesses several characteristic features and functions that are known to play important roles in HIV-1 replication and disease progression [40]. The presence of a functional Vpr in viral particles is necessary for the efficient translocation of the pre-integration complex (PIC) into the nucleus and subsequent infection of primary monocytes/macrophages and other non-dividing cells [5,6]. Vpr also has a crucial role in viral replication, apoptosis, cell cycle arrest and in the down-regulation of immune activation [37,41-43]. Many Vpr functions are carried out by virion-associated Vpr [44], suggesting that the incorporation of Vpr into virus particles is an important event not only in HIV-1 replication but also in HIV-1 mediated cyto-pathogenesis.

Several previous reports have indicated that p6 is phosphorylated during HIV-1 infection [2,22]. However, these studies did not undertake any detailed investigation of the biological significance of this phosphorylation event through biochemical or structural analyses. Our current computer-assisted structural modeling and AlphaScreen homogenous proximity assays have revealed that the phosphorylated Gag at Ser487 binds more stably to Vpr whereas there was no significant difference in the interaction of Gag-p6 with Alix, consistent with previous reports [31,32]. The phosphorylation of Ser487 can create another hydrogen bond between Gag-Ser487 and Vpr-Gln44. In consistent with this data a previous study indicated that the site specific deletion of Gln44 resulted in the significant reduction of Vpr incorporation into virions [34]. We also demonstrate that Gag phosphorylation at Ser487 affects Vpr incorporation and this process could be mediated by Gln44 residue of Vpr.

We show in our current study that Gag phosphorylation on Ser487 itself does not affect the binding affinity of Gag with Alix. However, resultant Vpr interaction to Gag may hinder the Alix-Gag interaction at the LYPXnL motif. This may eliminate Alix from nascent VLP and impeded its ability to function in HIV-1 release in PTAP-deficient strains of HIV. On the other hands, Alix also interacts with the nucleocapsid (NC) domain of HIV-1 Gag in addition to binding the LYPXnL motif [45], there by linking Gag to components of ESCRT-III. Therefore, further analysis is needed to fully understand the molecular link between Gag phosphorylation and virus release through the Alix/LYPXnL pathway.

We further explored the physiological significance of Vpr incorporation into virions. Our current results clearly demonstrate that the inhibition of aPKC-mediated Vpr incorporation prominently reduces the viral infectivity in MDMs. These results together indicate that Gag phosphorylation by aPKC plays a crucial role in the HIV-1 infection of macrophages.

aPKC has been demonstrated to be involved in cell polarity and migration in a number of study models [46]. During cell migration, aPKC localizes on the leading edge of the plasma membrane where HIV-1 Gag is also localized in infected cells. It has been reported in an earlier study that aPKC is located at an immunological synapse with potential importance in cell-to-cell viral transfer [47]. It is thus plausible that aPKC may regulate the incorporation of Vpr into virions at the leading edges or the HIV-1 virological synapse in polarized cells [48]. It would be interesting to investigate whether aPKC cooperates with other factors in polarized HIV-1-infected cells in an additional mechanism to its function in Gag phosphorylation.

In the earlier study by Folgueira et al. [49], it was demonstrated that aPKC mediates the NF-κB transcriptional activation required for HIV-1 infection in U937 cells. It is of particular interest that aPKC is a one of the key regulators of HIV-1 infection. Our present findings also provide evidence for the involvement of aPKC in HIV-1 replication by showing that it directly phosphorylates Gag on Ser487, and that this phosphorylation mediates Vpr incorporation into virions. The targeting of aPKC activity is therefore a potential option as a novel therapeutic intervention against HIV-1 infection in combination with existing anti-retroviral treatments.

## Conclusions

We have identified aPKC as a host protein kinase that phosphorylates HIV-1 Gag at its Ser487 residue. Computer assisted structural modeling and subsequent biochemical assays revealed that the phosphorylation of Gag Ser487 enhances the association of Gag with Vpr and promotes the resultant incorporation of Vpr into virions. These events facilitate viral infectivity in macrophages. Hence, aPKC inhibition is a potential new therapeutic approach against HIV-1 infection in human macrophages.

## Methods

### Viral DNA constructs and plasmids

The HIV-1 reporter virus vectors pNL4-3ΔEnv-Luc and pNL4-3ΔEnvΔVpr-Luc were provided by Akifumi Takaori-Kondo (Kyoto University, Kyoto, Japan) [50,51]. The HIV-1 recombinant molecular clone pHIV-1_89.6_ and pHIV-1_NLAD8_ were provided by Akio Adachi (Tokushima University, Tokushima, Japan). The HIV-1 Gag and HIV-1 p6 (pNL4-3) derived-DNA fragment was generated by PCR and inserted into the pEU-E01-GST-MCS vector (Cellfree Sciences, Yokohama, Japan). Using this subcloned plasmid, we generated substitution mutants with PrimSTAR Max (Takara Bio Inc, Shiga, Japan) and the following primers for Ser487A,

5’-TTAACTGCGCTCAGATCACTCTTTGGC-3’ and 5’-TCTGAGCGCAGTTAAAGGATACAGTTC-3’. Plasmids expressing HIV-1 Gag-Pol were provided by Jun Komano [52] (National Institute of Infectious Diseases, Tokyo, Japan). Expression vectors encoding aPKCλ wt and aPKCλ kn, a kinase-deficient mutant, have been previously described [53]. C-terminal Flag-tagged p55Gag (codon-optimized) has been previously described [54]. All the DNA experiments were approved by Gene and Recombination Experiment Safety Committee at the Yokohama City University School of Medicine.

### Antibodies and other reagents

The anti-p24 (CA) mouse monoclonal antibody (clone Kal-1) was purchased from Dako (Glostrup, Denmark). Anti-Flag (M2) and anti-Vinculin mouse monoclonal antibodies were obtained from Sigma (St. Louis, MO). Anti-PKCι mouse monoclonal antibody was from BD transduction (Franklin Lakes, NJ). Polyclonal rabbit anti-Vpr antibody was obtained from the AIDS research and Reference Reagent Program, National Institute of Allergy and Infectious Diseases (US National Institute of Health, Germantown, MD). The peptide specific antibody against PLT (pS) LRSLFGND (phosphorylated at Ser487 of Gag peptide from 484 to 495) was generated by Scrum Inc (Tokyo, Japan). The myristoylated (myr) PKC ζ peptide inhibitor myr-PKCζ (N-myr-Ser-Ile-Tyr-Arg-Arg-Gly-Ala-Arg-Arg-Trp-Arg-Lys-Leu-OH) and myr-PKC α and β (N-myr-Phe-Ala-Arg-Lys-Gly-Ala-Leu-Arg-Gln-NH_2_) were purchased from Merck (Darmstadt, Germany). Akt inhibitor was obtained from Calbiochem (Darmstadt, Germany), and the PI3K inhibitor Wortmannin was obtained from Merck. The Cdk inhibitor roscovitine was purchased from Promega (Madison, WI). All inhibitors were dissolved in DMSO and stocks were aliquoted and stored at −60°C until use. The final concentration of each inhibitor used is indicated in the figure legends.

### Cells and viruses

Monocytes were isolated from buffy coat from healthy blood donors by positive selection on Monocyte Enrichment Cocktail (Stemcell, Tukwila, WA) and Lymphoprep (Stemcell) density gradient centrifugation with SepMate-50 (Stemcell). MDMs were generated by culturing monocytes with 100 ng/ml granulocyte-macrophage colony-stimulation factor for 5 days. 293T and HeLa cells were cultured in DMEM (Gibco-BRL, Rockville, MD) supplemented with 10% (V/V) fetal bovine serum (FBS) (Gibco-BRL). HIV-1_89.6_ and HIV-1_NLAD-8_ strains were produced in 293T cells. Vesicular stomatitis virus G glycoprotein (VSV-G)-pseudotyped viruses were produced in 293T cells cotransfected with reporter virus plasmid and VSV-G using the calcium-phosphate method. The culture supernatants were collected and subjected to quantification of HIV-1 particle yields by p24CA antigen capture enzyme-linked immunosorbent assay (ELISA) (Zepto Metrix, Buffalo, NY). Monocyte isolation and treatment were approved by the Ethics Committee at the Yokohama City University School of Medicine.

### *In vitro* protein production

A total of 287 cDNAs encoding human protein kinases were constructed as described previously [23]. The protein production method has also been described previously [55-57]. Briefly, DNA templates containing a biotin-ligating sequence were amplified by split-PCR using cDNAs and corresponding primers, and then used with the GenDecoder protein production system (Cell Free Science, Ehime, Japan). For HIV-1 Gag protein synthesis, *Gag* genes derived from the pNL4-3 proviral plasmid [58] were generated by split-PCR, and used as template with a Wheat Germ Expression kit (Cell Free Science) in accordance with the manufacturer’s instructions.

### Alphascreen-based protein-protein interaction assays

AlphaScreen assays were performed as described previously [23]. All recombinant proteins used here was synthesized using a wheat germ based cell-free system as described above. For each protein kinase, 1 μl of crude recombinant biotinylated construct from the human kinase library was incubated with 1 μl of crude GST-Gag or GST-DHFR in 10 μl of kinase assay buffer (100 mM Tris–HCl pH8.0, 10 mM MgCl_2_, 0.1% Tween20, 0.1% BSA) at 37°C for 1 h in one well of a 384-well Optiplate (Perkin Elmer, Foster City, CA. In accordance with the AlphaScreen IgG (protein A) detection kit (Perkin Elmer) instruction manual, 15 μl of detection mixture containing 100 mM Tris–HCl pH 8.0, 0.01% Tween-20, 1 mg/ml BSA, 5 μg/ml Anti-FLAG antibody (GE healthcare, Buckinghamshire, UK), 5 ng streptavidin-coated donor beads and 5 ng anti-IgG (protein A) acceptor beads were added to each well followed by incubation at 26°C for 1 h. AlphaScreen signals from the mixture were detected using an EnVision device (PerkinElmer) with the AlphaScreen signal detection program.

### *In vitro* kinase assays

Biotinylated GST-Gag proteins were synthesized in wheat germ cell-free extracts as described above. The synthesized GST-Gag proteins were then purified using streptavidin conjugated magnet beads (Promega). The purified proteins on the beads were then incubated with recombinant aPKCiota (Cell Signaling Technology) in a 50 μl reaction mixture containing 20 mM Tris–HCl pH 7.5, 1 mM EDTA, 1 mM dithiothreitol, 150 mM NaCl, 5 mM MgCl_2_, 0.05% Tween-20, 100 μM ATP and 2 μCi [γ-^32^P] ATP. The reaction mixture was then incubated for 1 h at 37°C, and the products were subjected to electrophoresis on 10% SDS polyacrylamide gels and were detected with an image guider (BAS2500; Fujifilm, Tokyo, Japan).

### Western blotting

Cells were harvested at the indicated post-treatment time points with doxycycline, washed with phosphate-buffer saline (PBS), and treated with lysis buffer (0.02% sodium dodecyl sulfate [SDS], 0.5% Triton X-100, 300 mM NaCl, 20 mM Tris–HCl [pH 7.6], 1 mM EDTA, 1 mM dithiothreitol) for 20 min on ice. Multiple protease inhibitors, 200 μM sodium vanadate and 20 mM sodium fluoride were then added to the buffer. The samples were centrifuged at 18,000 g for 10 min at 4°C, and clarified cell extracts were assayed for protein concentration using a Bio-Rad kit. Equal amounts of proteins (20 ~ 50 μg) were resolved by SDS-10% polyacrylamide (acrylamide, 29.2; bisacrylamide, 0.8) gel electrophoresis (SDS-PAGE) in running buffer (250 mM glycine, 25 mM Tris, 0.1% SDS). The separated proteins were transferred to polyvinylidene difluoride membrane. The membranes were washed with blotting buffer (TBS containing 0.1% Tween 20) and blocked in 10% low-fat powdered milk in blotting buffer for 1 h at room temperature. Primary antibodies were added at appropriate dilutions in 3% bovine serum albumin in blotting buffer and rocked overnight at 4°C. The membranes were then further washed in blotting buffer and incubated with a horseradish peroxidase-conjugated secondary antibody at room temperature for 1 h. Target proteins were detected with an enhanced chemiluminescence detection system (GE Healthcare). Images were processed using Fluor Chem FC2 (Alpha Innotech Corp. Tokyo, Japan) with a cooled charge-coupled device (CCD) camera and assembled using Adobe Photoshop CS5 Extended.

### Identification of phosphorylation sites on HIV-1 gag by mass spectrometry

Samples were separated by SDS-PAGE (12.5%) and the gel was stained with Coomassie brilliant blue (CBB). Gag was excised from the stained gel and digested with trypsin in 50 mM NH_4_HCO_3_ (pH 8.0) for 12 h at 37°C. Phosphopeptides were enriched using Titansphere® Phos-TiO Kit (GLsciences, Tokyo, Japan), in accordance with the manufacturer’s instructions. The enriched phosphopeptides were then analyzed by MALDI-TOF/TOF-MS (4800 proteomics analyzer, AB SCIEX, Foster City, CA). The resulting raw MS spectrum was processed using the 4000 Series Explorer Software (AB SCIEX, Framingham, MA) to generate Mascot generic format. The obtained MS and MS/MS data were then searched against the SwissProt database (January, 2013; 538849 sequences) using Mascot version 2.4.1 software (Matrix Science, London, UK), to identify proteins and protein modification. The search parameters were as follows: trypsin digestion with two missed cleavages permitted, variable modifications (oxidation of Met and phosphorylation of Ser, Thr, and Tyr), peptide mass tolerance for MS data ±0.15 Da, and fragment mass tolerance ±0.3 Da. Phosphopeptides were determined primarily using the Mascot program and were confirmed manually through raw MS/MS sequence data checking for the neutral loss of the phosphate group (−98).

### Analysis of structural data and structural model construction

The 3D structure of Alix with wild-type Gag-p6 was predicted by homology modeling using Molecular Operating Environment (MOE) (Chemical Computing Group, Canada). X-ray crystal structure of Gag-p6-Alix (Protein Data Bank [PDB] code: 2R02) was used as template structure. Energy calculation was achieved with AMBER ff99 force field and the GB/VI implicit solvent energy function [59]. Next, on the basis of the predicted structural model of Alix with wild-type Gag-p6, 3D structures of Alix with Gag-p6S487A and phosphorylated Gag-p6-Ser487 were constructed using Molecular Builder in MOE.

3D structures of Vpr with wild-type Gag-p6, Gag-p6Ser487A, and phosphorylated Gag-p6-Ser487 were also predicted by docking simulations with ASEdock module in MOE, because of no complex structure of Gag-p6-Vpr. The complex structure was estimated with a nuclear magnetic resonance (NMR) structure of Vpr (PDB code: 1M8L [60]) and a NMR structure around helix II domain of Gag-p6 (Gag Y483 to S494) (PDB code: 2C55). Substitution and phosphorylation at Gag S487 were achieved with the Molecular Builder. Energy calculations in the docking simulations were achieved with the same force field as that for Gag-p6-Alix.

Finally, all of the constructed complex structures were thermodynamically optimized with energy minimization, to remove unfavorable steric contacts.

### Bimolecular fluorescence complementation assay (BiFC)

To detect interaction of Gag with Vpr, we used the BiFC technique. Briefly, two fragments of Kusabira-Green (KG) fluorescent protein are brought together by the interaction of two proteins fused to these fragments, thus allowing specific detection of interaction in living cells (Amalgaam). Vpr or Vpr Q44E were cloned into phmKGN-MN and Gag or GagSer487A into phmKGC-MC. 293T cells were cotransfected with 0.7 μg of the Vpr construct and 0.5 μg of the Gag construct. Two days post transfection, cells were harvested and then subjected to the flow cytometry for measuring BiFC signal as reported previously [34].

### Immunoprecipitation

Cells were lysed in Lysis buffer containing 50 mM Tris–HCl (pH 8.0), 150 mM NaCl, 1 mM EDTA, 1 mM DTT with Complete protease inhibitor cocktail (Roche Molecular Biochemicals, Indianapolis, IN) and PhosSTOP phosphatase inhibitor cocktail (Roche Molecular Biochemicals). Lysate were cleared by centrifugation at 12,000 × g for 15 min, followed by pull down using with anti-Flag M2 affinity Gel (Sigma). Samples were separated by SDS-PAGE and analysed by Western blot analyses.

### Single-cycle virus release assays

For infection-based assays, cells were infected with VSV-G-pseudotyped HIV-1 at an moi (multiplicity of infection) of 0.01 or 0.2 for eight hours and cultured for two days. In experiments using kinase inhibitors, cells were treated with each inhibitor at 12 h before virus infection. Virus-containing supernatants were harvested and filtered to remove cell debris, and viral p24 antigens were measured using an ELISA kit (Zepto Metrix). The cell lysates were prepared using HBST buffer (10 mM HEPES, pH 7.4, 150 mM NaCl, 0.5% Triton X-100) containing a protease inhibitor cocktail (Roche). Immunoblotting assays and the antibodies used have been described previously [61]. The culture supernatants and cell lysates were subjected to p24 ELISA or immunoblotting assays, as described above.

### HIV-1 production assay

Primary human macrophages were infected with HIV-1_89.6_ or HIV-1_NLAD-8_ virus. Two days post-infection, these cells were washed with PBS to eliminate the presence of virus. After washing, the cells were cultured either in media alone or media containing aPKC inhibitor. Infected macrophages were cultured for 12 days, during which time viral supernatants were collected and fresh media with inhibitors was also added every three days. The p24 levels contained in each viral supernatant sample was monitored using p24 ELISA (Zepto Metrix) in accordance with the manufacturer’s protocol.

## Competing interests

The authors declare that they have no competing interests.

## Authors’ contributions

AK, ST, HO, KM, AO and AI performed experiments. AK, ST, TS, SM, HK, WS, HS, HH, SO, NY and AR participated in the design of the study, the analysis of the data. AK and AR wrote the manuscript. All authors read and approved the final manuscript.
